# A Deep Learning-based Pipeline for Segmenting the Cerebral Cortex Laminar Structure in Histology Images

**DOI:** 10.1007/s12021-024-09688-0

**Published:** 2024-10-17

**Authors:** Jiaxuan Wang, Rui Gong, Shahrokh Heidari, Mitchell Rogers, Toshiki Tani, Hiroshi Abe, Noritaka Ichinohe, Alexander Woodward, Patrice J. Delmas

**Affiliations:** 1 Intelligent Vision Systems Lab, The University of Auckland, Auckland, New Zealand; 2 Theoretical Biology Group, Department of Creative Research, Exploratory Research Center on Life and Living Systems, National Institutes of Natural Sciences, 5-1 Higashiyama, Myodaiji, Okazaki, Aichi, 444-8787 Japan; 3 Laboratory for Molecular Analysis of Higher Brain Function, RIKEN Center for Brain Science, Wako, Japan; 4 Department of Ultrastructural Research, National Center of Neurology and Psychiatry, Tokyo, Japan

**Keywords:** Cerebral cortex laminar structure, Image segmentation, Deep learning, Artificial intelligence, Computer vision, Neuroscience, Neuroanatomy, Histological data, Cell microscopy

## Abstract

Characterizing the anatomical structure and connectivity between cortical regions is a critical step towards understanding the information processing properties of the brain and will help provide insight into the nature of neurological disorders. A key feature of the mammalian cerebral cortex is its laminar structure. Identifying these layers in neuroimaging data is important for understanding their global structure and to help understand the connectivity patterns of neurons in the brain. We studied Nissl-stained and myelin-stained slice images of the brain of the common marmoset (Callithrix jacchus), which is a new world monkey that is becoming increasingly popular in the neuroscience community as an object of study. We present a novel computational framework that first acquired the cortical labels using AI-based tools followed by a trained deep learning model to segment cerebral cortical layers. We obtained a Euclidean distance of $\varvec{1274.750 \pm 156.400}$ $\varvec{\mu m}$ for the cortical labels acquisition, which was in the acceptable range by computing the half Euclidean distance of the average cortex thickness ($\varvec{1800.630~ \mu m}$). We compared our cortical layer segmentation pipeline with the pipeline proposed by Wagstyl et al. (*PLoS biology,* *18*(4), e3000678 2020) adapted to 2D data. We obtained a better mean $\varvec{95^{th}}$ percentile Hausdorff distance (95HD) of $\varvec{92.150~ \mu m}$. Whereas a mean 95HD of $\varvec{94.170~ \mu m}$ was obtained from Wagstyl et al. We also compared our pipeline’s performance against theirs using their dataset (the BigBrain dataset). The results also showed better segmentation quality, $\varvec{85.318 \%}$ Jaccard Index acquired from our pipeline, while $\varvec{83.000 \%}$ was stated in their paper.

**Supplementary Information:**

The online version contains supplementary material available at 10.1007/s12021-024-09688-0.

## Introduction

Understanding the brain’s anatomical structure and how the brain processes information are critical steps to explore potential disease treatments (Baldassarre et al., 2016; Rowe, 2010). To this end, the neuronal connections between brain regions need to be studied further as they are vital to understanding neurological disorders (Minshew & Williams, 2007; Rowe, 2010). For example, tracing studies are one way that neuroanatomists can study how signals are being transmitted across the brain (Rockland, 2019). Connectivity patterns can be seen in other types of neuroscientific data such as, *DWI*-diffusion weighted structural MRI (Shamir et al., 2024), and the brain’s anatomical structures can be elucidated in spatial omics data analysis tasks (genomics) (Singhal et al., 2024), or are reflected in brain activity seen in techniques such as layer-fMRI. Understanding the connectivity patterns not just between regions, but also across the six cortical layers, is an important next step (e.g., as done in Vanni et al. 2020). Doing so can reveal the hierarchies of information flow between regions, showing feedforward and feedback projections, and aid in the elucidation and validation of theories of information processing in the brain. In a simplified description, signals from the thalamus project to cortical layer IV. Therefore, it has been defined as the primary input layer (Fröhlich, 2016), while layer V projects to both cortical and subcortical regions (Naidich et al., 2013).

In tracing studies, the six cortical layers cannot be seen in the fluorescent images taken of the brain (Woodward et al., 2020). Thus, the neuron cell connectivity between each cortical layer has to be studied afterward, for example, by overlaying the image of a Nissl-staining histological slice cut from the same brain (with the signal projection image). It is challenging and time-consuming for humans to manually identify the cortical layers in a large set of Nissl-stained images (e.g. over 200 images per brain and roughly 26000 $\times$ 21000 pixels per image). Therefore, using artificial intelligence techniques to automatically segment layers from the image would be helpful. To the best of our knowledge, there is no cortical layer annotation openly available for the common marmoset monkey brain, making it a worthy research goal to pursue.

Previous attempts, such as Kiwitz et al. (2020) first developed a modified convolutional neural network (CNN) motivated by the U-Net (Ronneberger et al., 2015) architecture to segment the human cortex areas hOc1 and hOc2 on the BigBrain dataset (Amunts et al., 2013). The cortex region segmentation results of this study were promising. Sub-layers in Layers III, IV, and VI were extracted and identified. However, the proposed process requires the extraction of intermediate outputs from the middle layer of the network manually, and only partial layers can be segmented. Wagstyl et al. (2020) utilized 1D profiles extracted from a 3D reconstructed brain volume. The profiles were fed to a 1D CNN to segment the laminar structure on silver-stained histological slice images. Recent advanced deep learning models, such as nnU-Net (Isensee et al., 2021), specifically target medical image segmentation tasks. Štajduhar et al. (2023) improved the cortical layer segmentation results further once again using the BigBrain dataset. All neurons were segmented using *k*-nearest neighbors (KNN) algorithm (Kumar et al., 2021) first, followed by analyzing the cell density. The authors identified Layer I and white matter regions given their small cell density. Lower cell density helped to identify layer II, while high cell density characterized layer IV. The proposed method was not automatic, and neuron cell segmentation only worked with the histological slice images containing non-overlapping neuron cells that can be individually identified.

Instead of segmenting individual layers, Zeng et al. (2023) developed a method to segment only the supragranular (Layer I-III) and infragranular (Layer V-VI) layers. They introduced U-Net-based semi-supervised learning combined with the cross-pseudo-supervision technique to deal with limited datasets. The authors used nnU-Net as the baseline for model evaluation and other state-of-the-art networks and showed better segmentation results of the supragranular and infragranular layers. Nevertheless, this study only used the annotation of a specific cortex region; other regions were not tested, which may lead to model bias towards a specific cortical area. Moreover, different brain morphology, different conditions, and parameter settings of the MRI images may also influence model performance. Lastly, this study only segmented the supragranular and infragranular layers, while our goal is to segment all six Brodmann layers.

Most of the methods have been tested on the BigBrain dataset, which was created based on the human brain, while other mammals, such as the common marmoset monkey brain, have been overlooked. This could be because only a few research groups are currently studying the common marmoset monkey brain; thus, computer vision scientists have not yet developed the AI-based tools required to analyze such data. Some studies discussed only used one evaluation metric (i.e., Jaccard Index), which shows the limitation of the performance measurement. Instead, we adapted seven metrics (including distance-based and overlap-based metrics) to measure the performance of our methods from different perspectives.

**Fig. 1:**
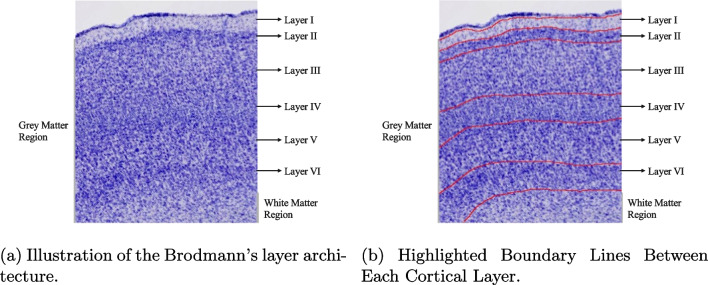
Brodmann’s layer illustration

Here, we developed a novel pipeline for segmenting Brodmann’s six cortical layers on Nissl-stain highlighted histological slice images (see Fig. 1). We focused on the laminae structure of the auditory cortex in the brain of the common marmoset monkey (*Callithrix jacchus*). This region was chosen due to its obvious importance for auditory information processing and its manageable size relative to the whole brain. It also serves as a starting point for applying our analysis to the entire brain in future work.

Our study has the following contributions:

- Multi-modal (Nissl-stained and myelin-stained) registered brain slices were used with ultra high-resolution of 9.2 $\mu m$ when compared to 20 $\mu m$ for the BigBrain dataset.
- We provided an AI-based algorithm for extracting the cortex area from the label images.
- We developed a fully automated end-to-end pipeline that performs Brodmann’s six cortical layer segmentation on the auditory cortex region.
- We developed a reference baseline of high-resolution cortical laminar segmentation in the healthy marmoset brain that can be used for future comparison to diseased brain data.

## Data

Three levels of the processed images derived from the original image set were employed. The key feature of our images is Brodmann’s Cortical Layer architecture. This study does not discuss regions (e.g., the white matter) deeper than Layer VI.

**Fig. 2:**
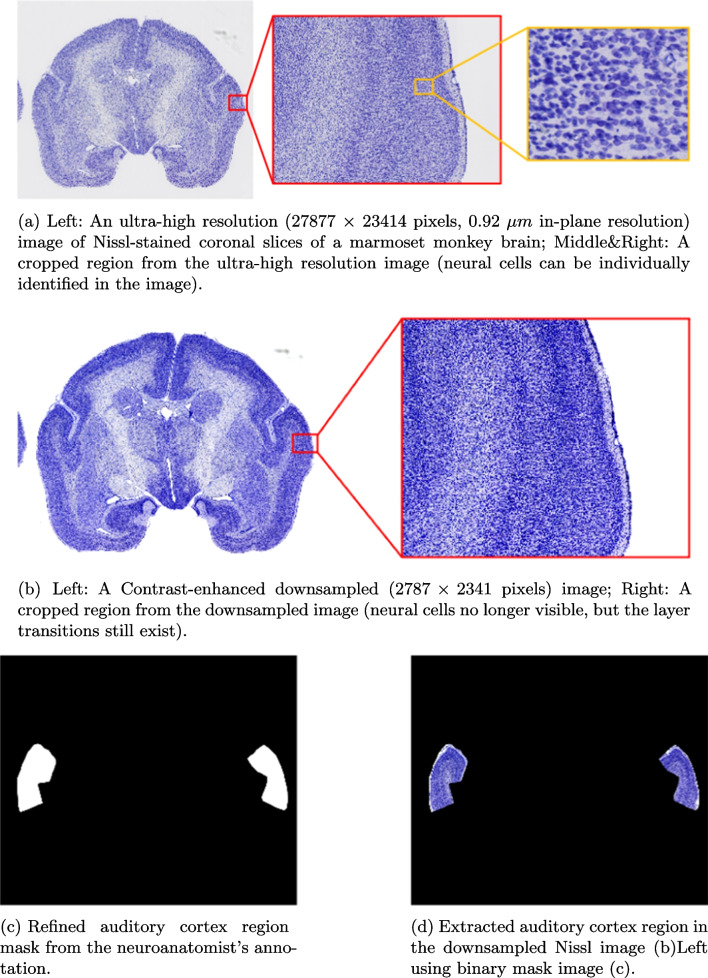
Examples of datasets

Three common marmoset monkey brains were used, which were previously utilized in physiological and anatomical studies of the auditory system (Abe et al., 2017). The brains were fixed in $4\%$ paraformaldehyde and sectioned coronally into 50 $\mu m$ thick slices using a freezing microtome (Yamato-Koki, Saitama, Japan). The slices were divided into three series: the first sections in each series were stained for myelin, the second sections were stained for Nissl substance with thionin, and the third sections were used for other purposes.

The first image processing level (Processing Level A) contains ultra high-resolution (roughly 26000 $\times$ 21000 pixels per image, 0.92 $\mu m$ in-plane resolution) images of Nissl-stained coronal slices of three common marmoset monkey brains (around 40 images per brain), they were captured by a NanoZoomer 2.0-HT batch slide scanner with 20$\times$ objective and a 455 nm per pixel resolution, where Nissl-stain highlighted neuron cells can be individually identified in the image (see Fig. 2a). However, during our early experiments, using ultra high-resolution images as the input to the deep learning model did not result in good segmentation results. This is likely because utilizing such large images results in more parameters for the model input layer. This may, in turn, increase the number of neurons for the subsequent hidden and output layers. Research suggests that deep learning networks with large number of parameters generalize well to the unseen data but usually require a large amount of training data and can be hard to train (Sarraf et al., 2021). Overfitting can be an issue with only 40 images per brain and only one brain for training our model. Observing that the transitions between layers are visually similar to a textural change (see Fig. 1b for illustration), we decided to downsample the ultra high-resolution images to be $10 \times$ smaller (roughly $2600 \times 2100$ pixels per image) than the original images and then enhanced the image contrast using Python Pillow^1^ library with factor value of 2.0, to better visualize the layer features in the images. The downsampled images (Processing Level B) no longer have the cell-level features. However, the cortical layer features in the image are still visible (e.g. see visible cortical layers transitions in Figs. 1b and 2b).

As we focused solely on the auditory cortex region, we leveraged neuroanatomist annotation to provide auditory cortex labels on myelin-stained highlighted histological slices obtained from adjacent Nissl slices (i.e. adjacent brain slices were used for myelin and Nissl staining). We then mapped these annotations into the slice image that has been applied with the Nissl stain. The created auditory cortex annotation images were labeled as Processing Level C (see Fig. 2c, d).

Other studies (Gao & Chen, 2011; Narayanan et al., 2020; Wagstyl et al., 2020) have used different staining techniques to visualize the cerebral cortex structure. For instance, the silver stain used in Wagstyl et al. (2020) highlights dendrites and axons (Alturkistani et al., 2015; Paul et al., 2008), and turns the histological section into a dark or black color, whereas our Nissl stain turns the section into a blue or light blue color.

**Fig. 3:**
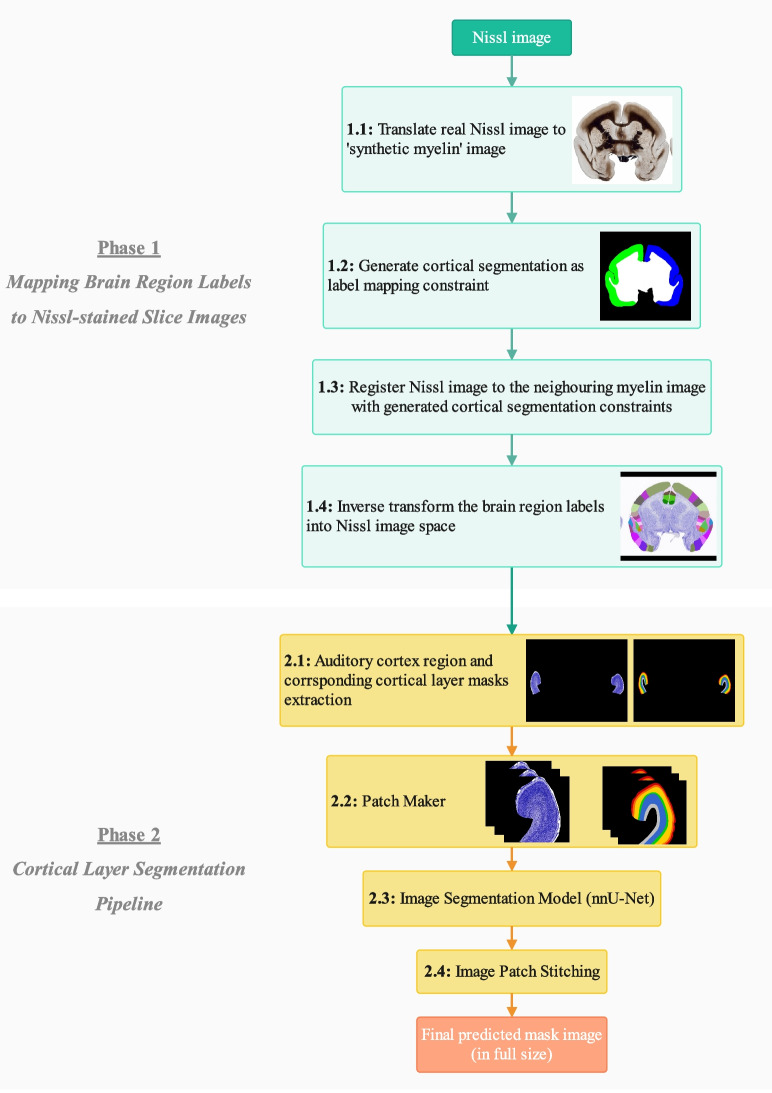
Proposed pipeline work flow diagram

## Methods

### Framework Overview

Our framework consists of two main phases. The first phase maps auditory cortex annotations from the image of the myelin-stained histological slice to the image of the adjacent histological slice with the Nissl-stain. The second phase focuses on segmenting the cortical layers in the auditory cortex region in the Nissl-stained images. Our workflow diagram is shown in Fig. 3. Detailed information for Phase 1 can be found in Section “Mapping Brain Region Labels to Nissl-stained Slice Images with AI Techniques (Phase 1)”, while Phase 2 is described in Section “Cortical Layer Segmentation Pipeline (Phase 2)”.

### Mapping Brain Region Labels to Nissl-stained Slice Images with AI Techniques (Phase 1)

The borders of the brain’s anatomical regions in the cortex were identified by neuroanatomists using myelin-stained histology data. To transform these anatomical region annotations on the myelin image, the Nissl image has to be aligned with its neighboring myelin image. This task proved challenging due to the difference in contrast and texture between myelin and Nissl-stained images. To help improve registration accuracy (Step 1.1), we used the AI-based algorithm CycleGAN (Zhu et al., 2020). CycleGAN provides unsupervised learning algorithms to translate Nissl to ‘synthetic myelin’ images - so that both images have similar contrast and texture.

To improve the registration quality even further, we decided to generate a cortical segmentation as a constraint during image registration (Step 1.2). This constraint has been used as an extra parameter when performing the image registration. We used *pix2pix* (Isola et al., 2018) to automatically generate a cortical segmentation on the ‘synthetic myelin’ images. The training data was manually drawn on the real myelin images by expert neuroanatomists.

To register a Nissl image to the neighouring myelin image (Step 1.3), we used the ‘antsRegistrationSyN.sh’^2^ script from the Advanced Normalisation Tools (ANTs) toolkit (Avants et al., 2009). This script registers each Nissl image to the neighboring myelin image (e.g., Nissl slice no. 3 to myelin slice no. 2) with cortical segmentation constraints, using a combination of linear (rigid and affine) and nonlinear (SyN) transformations. After the image registration step (Step 1.3), an inverse transformation (Step 1.4) was performed by using another script from the ANTs (‘antsApplyTransforms’^3^) to map the region labels in myelin image space to Nissl image space, thus giving region label overlays for Nissl images. These region labels can then be used to conduct our layer analysis of the auditory cortex, described below.

### Cortical Layer Segmentation Pipeline (Phase 2)

### Auditory Cortex Extraction

From Phase 1, we now have region label overlays for Nissl images. Since each region is labeled with different pixel intensity values (or brain region ID), in Phase 2, Step 2.1, we extracted only the auditory cortex region by utilizing the label of cortex region pixel intensity lookup table. The brain region IDs were defined using the Brain/MINDS 3D digital marmoset atlas (Woodward et al., 2018). Because our data is at a higher resolution than the region label overlays, we manually edited the mask boundaries using the GIMP image editing software (Peck, 2006). Based on the masks obtained, we extracted the auditory cortex regions from the left and right hemispheres in the Nissl-stain highlighted slices.

**Fig. 4:**
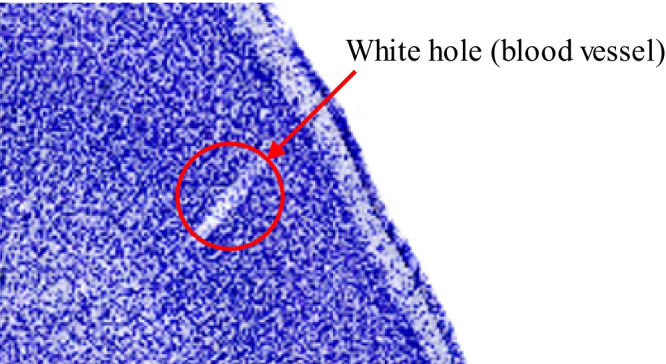
An example of a white hole in the Nissl image

**Fig. 5:**
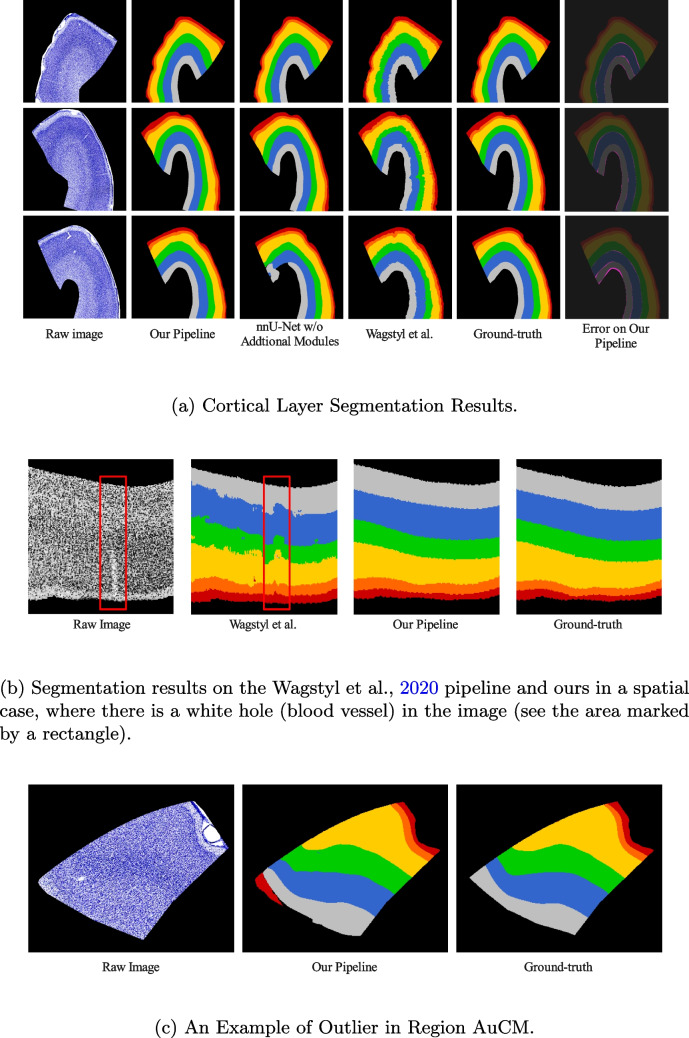
Segmentation results. Layer I: red; Layer II: orange; Layer III: yellow; Layer IV: green; Layer V: blue; Layer VI: grey; Error: purple

### Patch Maker

Training a deep learning model usually requires a lot of training data; otherwise, the model may overfit the training set and not generalize well to the new dataset. However, the model overfitting problem could be challenging since this study has only about 40 training images per brain available. A study developed by Wagstyl et al. (2020) used 1D profiles extracted from the 3D raw image across the cortical region, which created around ten thousand training samples. However, nnU-Net does not take 1D profiles as the input to the network; moreover, due to the nature of our Nissl images (i.e., small white holes (blood vessels) may appear in the auditory cortex region, see Fig. 4), the 1D convolution neural network may create some artifacts around those areas (see Fig. 5b). Lastly, using 2D images as the input sample removes the profile extraction phase completely. Instead, we adapted an overlapping patches approach to increase the number of training samples (see Step 2.2). The use of overlapping patches in the patch-wise U-Net approach is considered a successful data augmentation strategy that improves the model performance by reducing boundary artifacts and increasing contextual information around the features of interest, which is known to prevent overfitting. Further, the ability to aggregate data through overlapping patches around features statistically reduces variance. Our research followed the overlapping patches strategy introduced in Lee et al. (2020). This study compared the performance of using overlapping and non-overlapping patches on MRI data as the training set. Results showed that when using the Dice Coefficient and Jaccard Index metrics, a model trained with overlapping patches performed better than with non-overlapping patches.

This approach is similar to the sliding window technique; the window size was set to $512 \times 512$ pixels, and the stride was set to 32; this allows different patches to capture the same region of the image several times for increasing the training set size. The $512 \times 512$ pixels window size was big enough for the model to learn the locations of the cortical layers since the majority of the patch contains all the cortical layer features. Due to the entire image being partitioned into overlapped patches, there might be some situations where one patch loses the contextual information of the six-layer cortex. Hence, a patch filter mechanism was developed to ensure the quality of the patches in the training set. The filter checks the following three conditions for each pair of patches:

1. The mask patch has to contain all six layer labels.
2. Each layer label in the mask patch has to have at least 5 pixels.
3. The mean pixel intensity value for each feature patch has to be greater than 10.

Any pair of patches that does not satisfy the above criteria was removed from the training set.

### Image Segmentation Model

The next step in the pipeline is the Image Segmentation Model (Step 2.3), which feeds patches to the nnU-Net for training. The nnU-Net core segmentation network uses a U-Net architecture (Ronneberger et al., 2015) with some modifications. Such a network utilizes skip-connections (Drozdzal et al., 2016), which allow the model to learn the spatial information in the image more easily compared to a CNN. Furthermore, nnU-Net adds a deep supervision module (Li et al., 2022), adding additional losses to the two lowest-resolution layers in the expansion path. This allows for the shallower layers to learn more meaningful representations of the features in the image and accelerates the model convergence towards the global minimum (Li et al., 2019; Ren et al., 2023).

The nnU-Net self-configuration module was set up for common public datasets. However, it may not be helpful with our own dataset. Thus, we manually tuned some of the hyper-parameters to better fit our dataset and goals, which resulted in better segmentation performance. For instance, we prevented nnU-Net from automatically cropping the image into small patches, as we have our own patch-making mechanism.

Nissl-stain highlighted histological slices of three different common marmoset monkey brains were used. One brain for training the model, one for testing, and one for validation. For more information on the deep learning model and hyper-parameter setting, please see the Supplementary Information.

### Image Patch Stitching

The model takes 512$\times$512 pixels images, and the new unseen images also need to be cropped to be fed to the network for inference. Thus, the last step (Step 2.4) in the pipeline is to stitch these overlapping predicted mask patches back together to make one final output mask. The intuitive way is to copy each pixel in the predicted mask patch to the new mask image with the same size as the original Nissl-stained image. Nevertheless, even if a patch filter is used, the prediction of a few patches may not be good due to the poor position cropped from the original image; copying such images may result in overriding the pixels that are actually correct. Hence, an image interpolation mechanism was used to interpolate each pixel in the final predicted mask image from multiple patches, not just one. This image interpolation mechanism creates six heatmaps, one heatmap represents one cortical layer prediction.

Given the nature of the heatmap image, which exhibits a bimodal distribution, it was decided to apply an automatic locally adaptive thresholding technique (Roy et al., 2014) to the heatmaps. All pixels below the threshold were set to black. All non-zero pixels within the heatmap were set to a predetermined value for each layer, followed by merging to obtain an image of multi-layer masks with the original image size.

### Implementation

The Python version used to develop our framework was 3.11.4, and the Pytorch version was 2.0.1 with CUDA version 11.8. The nnU-Net package was downloaded from the GitHub repository^4^ with version number 2.2. Our solution was developed and tested on Windows 11 platform, and used the Windows Command Prompt to interact with the nnU-Net module.

The auditory cortex mask template acquired from the cortex mapping process was manually refined in a generic image editing software. As we discussed previously, two different resolution Nissl image sets from Processing Levels A and B were used. However, annotating the cortical layers in the high-resolution images from Processing Level A was time-consuming, and during our early experiment, the deep learning model using high-resolution images as the training data yielded worse results than the one using low-resolution images. Thus, we decided to annotate the layers as well as the auditory cortex mask on the low-resolution images.

### Model Training and Evaluation Method

Due to the limited dataset (three common marmoset monkey brains, $X_1, X_2, X_3$, were used, they all came from the same dataset domain), a nested 3-fold cross-validation was used. The outer cross-validation consisted of a training set containing two brains ($X_1$ and $X_2$) and a test set containing one brain ($X_3$). We further split the two brains in the training set for inner cross-validation. We trained two models using $X_1$ and $X_2$ and chose the model in the inner cross-validation with the best performance tested on the validation set. This selected model was then used to evaluate the final performance on the test set, $X_3$. For each iteration of the outer cross-validation, we swapped the brain used in the test set with the one used in the training set or the validation set. The nested cross-validation and data-splitting methods ensured each brain was used for training, validation, and testing, and the data in each set was independent of each other.

While mentioning the brain in the previous paragraph, each brain consisted of around 40 Nissl images. After passing through the Patch Maker module, more than 2,000 patches per brain were generated and fed to the model for training, and the same number of images were generated for the validation set and test set.

Since our pipeline consists of two main phases (see Sections Mapping Brain Region Labels to Nissl-stained Slice Images with AI Techniques (Phase 1) and Cortical Layer Segmentation Pipeline (Phase 2)), we performed the evaluation in two steps. The first step was to evaluate the performance of mapping brain region labels from the myelin image to the Nissl image. Because of the complex internal structure of the brain, we needed to find structures that can be used as anchors. Fortunately, there are a number of blood vessels going through the cortex region, creating white holes in the image. The locations of the white holes in the original image should be the same after the transformation. Thus, we used the center point of the white hole in the two images as the anchor. We calculated the position shift distance (the Euclidean distance) of the two anchor points in the original image and the transformed image as the mapping accuracy. The higher the distance value, the worse the mapping quality, and vice-versa.

For the second step of the evaluation, we employed four commonly used overlap-based image segmentation metrics, as per literature review guidelines (Maier-Hein et al., 2024; Müller et al., 2022; Yeghiazaryan & Voiculescu, 2018): the Jaccard index or Intersection-over-Union (IoU) (1), the Dice coefficient (DSC) (2), Recall, and Precision. These metrics were used to assess the pipeline performance against manual annotations verified by the information stated in a few cortical layer reference papers (Atapour et al., 2018; Palomero-Gallagher & Zilles, 2019) and an expert neuroanatomist.

1 $$\begin{aligned} IoU = \frac{|y \cap \hat{y}|}{|y \cup \hat{y}|} = \frac{1}{N} \cdot \sum ^N_{i=1} \frac{y_i \times \hat{y}_i}{y_i + \hat{y}_i - (y_i \times \hat{y}_i)} \end{aligned}$$

2 $$\begin{aligned} DSC = \frac{2 \times |y \cap \hat{y}|}{|y| + |\hat{y}|} = \frac{1}{N} \cdot \sum ^N_{i=1} \bigg ( \frac{2y_i\hat{y}_i + 1}{y_i + \hat{y}_i + 1} \bigg ) \end{aligned}$$

We denote N as the class number to predict, $y_i$ as the ground truth label and $\hat{y}_i$ as the predicted label for $i^{th}$ class. We calculated the average Jaccard index across six layers. The higher the average Jaccard index, the better the segmentation quality. The same is true for the Dice coefficient, Recall, and Precision.

Distance or contour-based image segmentation metrics: the average Hausdorff distance (AHD) (Müller et al., 2022), $95^{th}$ percentile Hausdorff distance (95HD) (Celaya et al., 2024) and average symmetric surface distance (ASSD) (Heimann et al., 2009) were also employed. These metrics compute the position shift of the layer boundaries in the predicted images compared to the label images. The Hausdorff distance (HD) (Maier-Hein et al., 2024) measures the maximum distance between two layers, where the reference points on each layer have the minimum distance from each other (see Eq. 3). However, it is known to be highly outlier-sensitive, so the AHD is usually used (see Eq. 4); it averages all distances between two reference points rather than returning the maximum distance value. The 95HD is even more robust to outliers by only sampling 95 percent of the data over the entire population. Lastly, the ASSD calculates the average distance of all the points on the predicted mask boundary to the boundary of the label images, and vice-versa (see Eq. 5). All distances ($\Vert \cdot \Vert$) were calculated using the Euclidean distance.

3 $$\begin{aligned} HD(A, B) = \underset{a \in A}{\max }\ \{\underset{b \in B}{\min }\ \Vert a-b\Vert \} \end{aligned}$$

4 $$\begin{aligned} AHD(A, B) = \frac{1}{N} \underset{a \in A}{\sum }\ \underset{b \in B}{\min }\ \Vert a-b\Vert \end{aligned}$$

5 $$\begin{aligned} ASSD(A, B) = \frac{1}{|A| + |B|} \left( \underset{a \in A}{\sum }\ \underset{b \in B}{\min }\ \Vert a-b\Vert + \underset{b \in B}{\sum }\ \underset{a \in A}{\min }\ \Vert b-a\Vert \right) \end{aligned}$$

We denote two sets of points in each of the layers as A and B, while a and b represent the reference points on each layer. We determined that, the accuracy of the layer boundary location predictions is more important than the amount of the overlapping area between the prediction and ground-truth (which measured by the overlap-based metrics). Since we aim to automatically generate cortical layer masks that can be overlayed with fluorescent images to see the neuron transmission between cortical layers, inaccurate layer boundary position prediction could lead to incorrect interpretation of the neuron transmission patterns, which subsequently affects other neuroscience studies. Overlap-based metrics are not sensitive to boundary errors (Taha & Hanbury, 2015). Thus, we used a distance-based metric (95HD) as the primary evaluation method. The 95HD is more robust to outliers than the vanilla HD and AHD but still gives the maximum errors over the 95 percent of the population, whereas ASSD only gives the overall errors. The overlap-based metrics are provided as references.

**Table 1:** Segmentation performance comparison

Metric	Wagstyl et al.-2D	nnU-Net w/o additional modules	Our pipeline
IoU	0.723±0.141	0.825±0.030	
DSC	0.795±0.141	0.903±0.018	
Recall	0.800±0.153	0.912±0.020	
Precision	0.816±0.123	0.898±0.020	
AHD ($\mu m$)	**95.400**	585.960	
95HD ($\mu m$)	94.170	138.970	
ASSD	3.774±1.819	2.341±0.875

**Table 2:** Segmentation performance on each layer

Layer number	Layer I	Layer II	Layer III	Layer IV	Layer V	Layer VI
IoU	0.814	0.808	**0.922**	0.888	0.918	0.879
DSC	0.897	0.892	**0.959**	0.939	0.957	0.935
Recall	0.846	**0.964**	0.941	0.961	0.941	0.947
Precision	0.956	0.831	**0.979**	0.920	0.973	0.923
AHD ($\mu m$)	2707.490	1189.400	**345.710**	660.930	1324.350	1821.370
95HD ($\mu m$)	160.780	**46.000**	65.050	110.460	294.550	598.310
ASSD	1.468	1.209	**1.194**	1.531	1.689	2.040
Thickness ($\mu m$)	92.200	80.620	247.390	186.820	338.380	177.200

## Results

### Mapping Brain Region Labels

In the first part of the pipeline, we aimed to map the cortex labels from the myelin image to the Nissl image (see Fig. 3, Phase 1). In order to measure the performance of the mapping algorithm, we used the Euclidean distance discussed above. We defined the acceptable range for the mapping as the half Euclidean distance of the average cortex thickness (1800.630 $\mu m$) across the whole brain. If the mapped cortex labels have an offset distance larger than the half thickness of the cortex, it means the labels did not overlap well with the cortex region.

We achieved an Euclidean distance of $1274.750 \pm 156.400$ $\mu m$ for the brain cortex region label mapping phase, which is within our defined acceptable range. Since the center point of the white holes was selected manually by humans, we may have measurement errors (i.e., the selected points are not exactly centered). The errors were measured by calculating the diameter of the white hole along the long side. Since the white holes in the image are very small, the measurement errors are acceptable.

### Cortical Layer Segmentation

For the second half of the pipeline, we obtained a performance metric (see Table 1). Our proposed pipeline (marked in  in Table 1) achieved a mean 95HD of 92.150 $\mu m$, a mean IoU of 0.872±0.043, and a mean DSC of 0.930±0.025 on the test set. These results indicate that our pipeline can accurately segment Brodmann’s Six Cortical Layer. Figure 5a shows examples of cortical segmentation. Furthermore, the model performance was also evaluated per each layer (see Table 2). Layer II had the lowest 95HD=46.000 $\mu m$, while Layer VI had the highest 95HD=598.310 $\mu m$. The best performance of each metric is highlighted in **bold**.

**Table 3:** Auditory cortex region names

Abrreviation	Definition	Abrreviation	Definition
AuAL	Anterolateral area	AuR	Rostral area
AuCL	Caudolateral area	AuRPB	Rostral parabelt
AuCM	Caudomedial area	AuRM	Rostromedial area
AuCPB	Caudal parabelt area	AuRTL	Rostrotemporal lateral area
AuML	Middle lateral area	AuRTM	Rostrotemporal medial area
AuA1	Primary area	AuRT	Rostrotemporal part

**Fig. 6:**
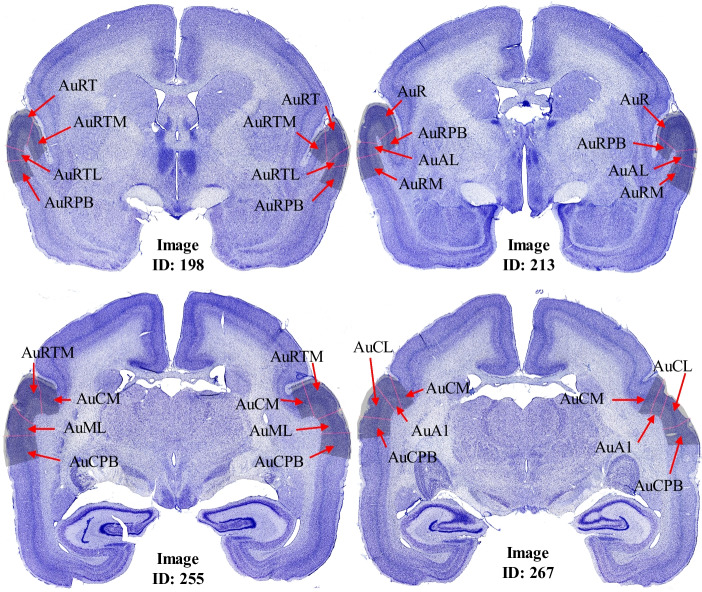
Location map of the auditory cortex regions. Note: one slice may not contain all the auditory cortex regions since some of them only appear close to the front of the brain and disappear when moving towards the back, while other regions may only appear on the back of the brain. The above examples were selected from the dataset to show different regions in slice images

When comparing different segmentation pipelines using distance-based metrics, the AHD and 95HD suggested different stores. Recall the discussion between these two metrics in Sec. “Model Training and Evaluation Method”; outliers may occur in our results since the AHD still includes outliers in the computation. A further analysis was conducted. For some regions in the auditory cortex, the texture transition across the layers can be more subtle than in other regions. Thus, we split the auditory cortex region into several small sub-regions. The auditory cortex can be split further into 12 auditory cortex regions (Stephan et al., 2012), for example, the Anterolateral Area (AuAL), the Caudolateral Area (AuCL), the Caudomedial Area (AuCM). See Table 3 for the full definition of the auditory cortex regions, and Fig. 6 for the location of each region.)

**Table 4:** Segmentation performance per auditory cortex region

Region name	IoU	DSC	Recall	Precision	AHD ($\mu m$)	95HD ($\mu m$)	ASSD
AuAL	0.862	0.923	0.928	0.925	85.480	36.690	0.942
AuCL	0.896	0.943	0.947	0.943	143.900	51.150	0.895
AuCM	0.895	0.943	0.947	0.942	464.780	317.070	1.718
AuCPB	0.866	0.924	0.920	0.935	326.640	182.030	1.811
AuML	0.909	0.951	0.951	0.954	**48.550**	**31.090**	**0.641**
AuA1	**0.921**	**0.958**	**0.961**	**0.959**	232.370	125.330	0.794
AuR	0.853	0.918	0.929	0.917	229.500	75.770	1.439
AuRPB	0.826	0.903	0.915	0.901	97.380	64.560	1.575
AuRM	0.842	0.910	0.914	0.914	205.430	88.940	1.446
AuRTL	0.821	0.899	0.900	0.905	64.460	46.860	1.164
AuRTM	0.804	0.889	0.899	0.888	189.870	117.220	1.676
AuRT	0.815	0.897	0.907	0.900	141.020	82.470	1.703

For each metric, the best performance is highlighted in **bold**. See Table 3 for auditory cortex regions’ name definition

The same evaluation metrics were applied to each auditory cortex region. Table 4 shows the model performance on each individual region. Due to the computational differences between the overlap-based metrics and distance-based metrics, performance disagreement occurred. Using distance-based metrics, the AuML region had the best prediction result, whereas the AuCM region had the worst result, but the overlap-based metrics showed otherwise. This result indicated that there were a few outliers in the predicted images. Although the model performance on one or two regions was not expected, the model’s overall performance is good enough for further neuroscience-related studies.

To show the effect of the Patch Maker and Patch Stitcher modules, we trained a separate model only using the full Nissl images and standard nnU-Net configuration. For this model, the 95HD was 138.970 $\mu m$ (> 92.150 $\mu m$, which was obtained from the proposed pipeline), indicating that the two modules we developed increased the model performance.

We compared our pipeline with a previous study developed by Wagstyl et al. (2020), which focussed on 3D data. First, we modified their pipeline to work on 2D data. We call this pipeline Wagstyl et al.-2D, as the baseline model. Table 1 provides the model performance comparison. The baseline model was developed based on their paper and source code and has been verified by training and testing the model with the 2D dataset provided in their paper. The 95HD and IoU obtained from their model on our dataset were 94.170 $\mu m$ and 0.723±0.141, whereas 92.150 $\mu m$ and 0.872±0.043 were obtained from our pipeline, respectively. We tested the statistical significance of our results by using the non-parametric Mann-Whitney U test (or Wilcoxon rank-sum test) (MacFarland & Yates, 2016) and Shapiro-Wilk test (Ghasemi & Zahediasl, 2012). We chose the Mann-Whitney U test because our data does not come from the normal distribution (all Shapiro-Wilk test p-values were smaller than 0.05, which rejects the null hypothesis, stating that the data was not normally distributed). We report the significance of the differences for each fold of the 3-fold cross-validation. The p-values of the Mann-Whitnet U test on each fold were 0.010, $1.545 \times 10^{-6}$, and $1.545 \times 10^{-6}$. All p-values were smaller than 0.05, which rejects the null hypothesis that states the true mean of the 95HD value is equal between the two groups of data. Thus, we concluded that, for each fold, the 95HD differences between the two groups of data were statistically significant. For the IoU value comparison, the Mann-Whitney U test was also used since, for every fold, the two groups of data were not normally distributed (all Shapiro-Wilk test p-values were smaller than 0.05). The p-values of the Mann-Whitney U test on each fold were $1.508 \times 10^{-6}$, $1.542 \times 10^{-6}$, and $1.542 \times 10^{-6}$. All p-values obtained were smaller than 0.05, which rejects the null hypothesis that states that the true mean of the IoU score is equal between the two groups of data. Thus, we have enough evidence to state that, for each fold, the IoU score differences between the two groups were statistically significant. A visual comparison between our and Wagstyle et al.-2D pipelines can be found in Fig. 5a.

## Discussion

We validated our results by upsampling the borders to higher-resolution images and comparing these to expert neuroanatomists’ estimated borders. Both borders were found to be visually similar.

**Fig. 7:**
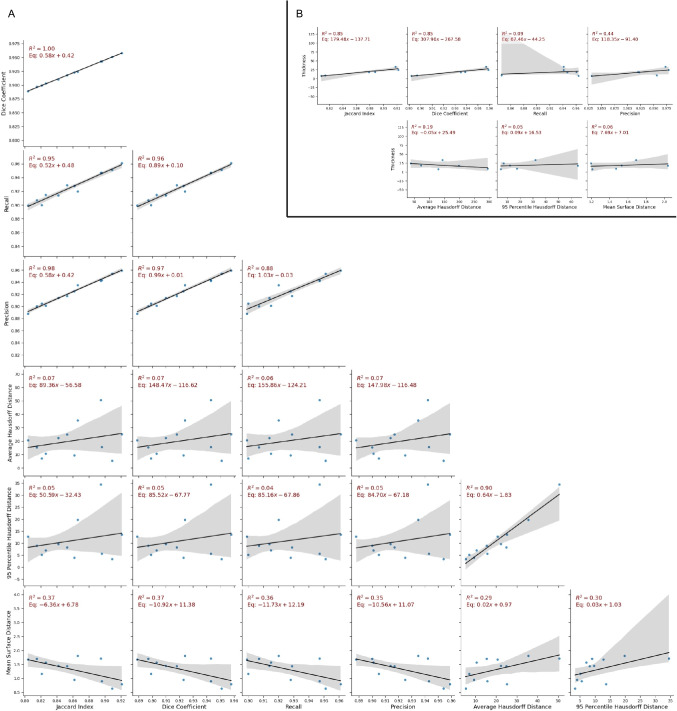
Correlation Plots. A: Correlation plots between different evaluation metrics in different auditory cortex regions. B: Correlation plots between the segmentation performance and cortical layer thickness measured using Euclidean distance

We noticed the difference in the model performance on each cortical layer. This may be caused by the clearness of the layer boundaries. A clear layer transition (i.e., Layer I and Layer II, see Fig. 1b) would result in a low 95HD value compared to other layer boundaries. The thickness of the cortical layer may also influence the model performance, as the more pixels involved in describing the feature in the image, the better the model understands the content of the features. Figure 7B indicates a strong correlation ($R^2 = 0.850$) between each layer thickness and its IoU and DSC. When using boundary-based metrics, the clearness of the transition between two layers becomes more important than the thickness of the layer itself, since a clear transition reduces the uncertainty of the boundary location.

No correlation between the layer thickness and recall was observed. For example, Layer II had a slightly low Jaccard index and precision but a high recall value. This suggests that the model may have over- or under-segmented some areas, which caused a slightly lower Jaccard index and precision. However, due to the fact that most of the area was segmented, the recall value is high. One reason could be that when visualizing layer’s transitions (see Fig. 1a), the transition between Layer II and Layer III is smoother than other layers’ transitions. This leads to difficulties for the model to identify the exact layer boundary, thus, layer thickness does not affect the recall and precision values.

The model performance disagreement occurred when comparing the AHD and other metrics when assessing the performance of each region (see metrics correlation plot in Fig. 7A). This may indicate that outliers may occur in our results. Figure 5c shows an example of the outlier in the AuCM region, which has the highest AHD, while the overlap-based metrics are in the acceptable range. Due to the similar textural color between Layer I and the border region of Layer VI, the model may misidentify these two layer features, causing incorrect segmentation. This kind of result drastically increased the AHD, while no significant changes were found in the overlap-based metrics. Since the rough locations of the cortical layer are well-known to expert neuroanatomists, the outliers can be easily identified and disregarded. Therefore, the outlier predictions are still usable.

**Fig. 8:**
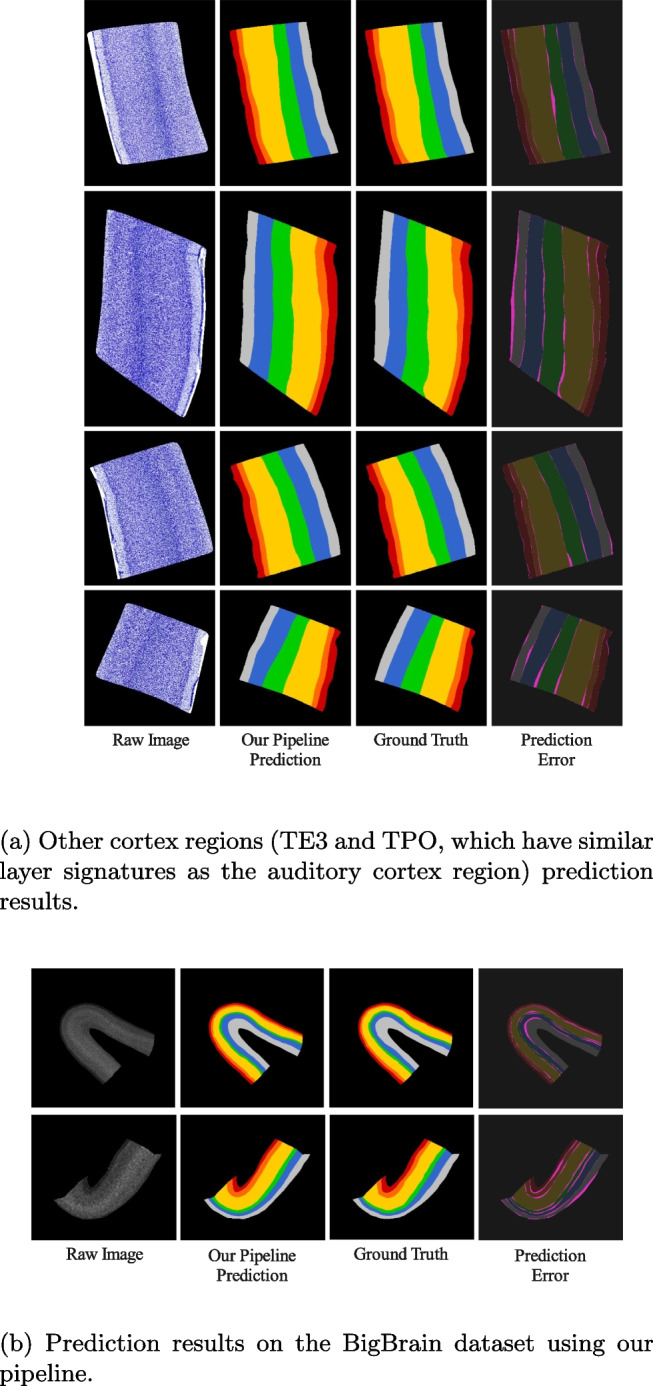
Segmentation results

We compared our pipeline with the one proposed by Wagstyl et al. (2020). We adapted their pipeline from 3D data to 2D (Wagstyl et al.-2D) as the baseline for comparison applied with our dataset. Our pipeline achieved better cortical layer segmentation results when compared with Wagstyl et al.-2D. By comparing model architecture, we used a more advanced medical image segmentation model (nnU-Net) when compared to the generic 1D CNN used by Wagstyl et al. (2020). nnU-Net used the U-Net architecture, which includes the skip connection module. This module improves the segmentation quality by copying the spatial information of the shallow layers to the deep layers so that the loss of the localization details in the image is mitigated (Drozdzal et al., 2016; Ronneberger et al., 2015).

A 2D convolutional operation in nnU-Net considers the spatial information in both dimensions, whereas in 1D convolution, only one dimension is considered. Figure 5b shows a special case in our image dataset. There is a blood vessel going through the histology slice, which is not being stained as the Nissl stain does not color the blood vessel. In this situation, the Wagstyl et al.-2D pipeline created an artifact, as shown in Fig. 5b, while this special case did not affect our pipeline.

Further testing was undertaken to show the potential of our pipeline in predicting cortical layers in other regions (not part of our training set). Figure 8a indicates our pipeline can predict more than just the cortical layers on the auditory cortex. A good segmentation of the TE3 and TPO cortex suggests that the segmentation model can be used for predicting cortical layers in other cortices, given that the layer features of that cortex are similar to the auditory cortex. We trained another model with the BigBrain dataset (see Fig. 8b) and achieved better segmentation quality (Jaccard Index = 85.318%) on their data when compared to the accuracy stated in their paper (83.000%). As their paper only used the Jaccard index, comparisons of other metrics are not discussed.

### Limitations

Our framework demonstrates good segmentation quality on our down-sampled Nissl-stain histological slice images. However, a few limitations were observed. As our pipeline was only tested on three brains, our results may not generalize well to the more diverse range of brains.

The mapping of the auditory cortex annotation from the myelin-stained image to the Nissl-stained image was not always accurate. This could be due to damage or artifacts in the myelin and Nissl images, causing difficulties when performing image registration and inverse transformation using ANTs. Fortunately, the feature quality was not affected outside the damaged area, which only represents roughly 0.004% of the total slice area. Therefore, we had to manually edit the image generated by the model so that it did not affect the next step in the pipeline.

Our pipeline suffers if the input image does not contain enough layer features. For example, the boundary between Layer II and III is unclear, allowing slight over- and under-segmentation to occur. The thickness of the histological slice may also affect the image’s appearance. The thicker the slices are, the more neuron cells that are highlighted by the stain, which introduces more noise into the image. However, this only affects traditional segmentation algorithms since they struggled with out-of-focus cells in the Nissl image. The neural network approach can handle both in-focus and out-of-focus cells.

Our pipeline was only trained on the auditory cortex, which has a distinct six-layer pattern. Using this pipeline with cortices that do not have a six-layer pattern may lead to incorrect cortical layer segmentation results.

Due to the histological slices being stained manually by expert neuroanatomists, uneven stained slices may occur, leading to a slightly less clear layer transition in the image, which may affect the model performance.

As only 2D data was used, while Wagstyl et al. (2020) used 1D profiles extracted from 3D data, a comparison between the two studies may not be perfect, but since our histological slices around the auditory cortex region, which were cut close to being along the columnar direction across the cortical layers, the 1D profiles extracted from the images are very similar to the profiles extracted from 3D, thus, the differences are negligible.

Despite the above limitations, our pipeline still produced good segmentation results, and we expect it to be used for future neuroscientific studies, such as in anterograde tracing studies.

## Conclusion

In this paper, we proposed a novel pipeline for segmenting cortical layers in images Nissl-stain highlighted histological slices in 2D. We compared this to the modified 2D version of the Wagstyl et al. (2020) pipeline. Our proposed pipeline performed better than the modified version of their pipeline as measured by several commonly used metrics. Cross-testing of our pipeline was completed by training a new model on the BigBrain dataset. Our pipeline performed better on the BigBrain dataset than what was stated in their paper. Our pipeline was further tested on additional cortex regions and datasets as discussed in Sec. “Discussion”. It was found that our original model could perform on other regions of the cortex as long as the layer signatures of these regions remained similar to the auditory cortex.

Possible future research may include fine-tuning the hyperparameters of CycleGAN and pix2pix to increase the region label mapping accuracy (Gudavalli et al., 2024; Tsuda & Hotta, 2019) and avoid additional manual brain cortex region label refinement.

When considering the possible use of 3D datasets of cortex regions, it was found that the required resolution to access the cell level can only be achieved through a brain-slicing mechanism, which, in essence, is a 2D data process (regardless of the slice thickness). Therefore starting from 2D data and delineating the layers has value. A way to address issues with downsampling, which may lead to inaccurate border detection, would be to adopt an iterative downsampling technique. This technique would allow us to use multi-scale pyramids (from high-resolution to low-resolution images) as the training data, leading to better cortical layer segmentation quality. Given the difficulty of accessing or creating (from 2D slices) 3D brain data at the required resolution, exploring metrics comparing 2D to 1D profiles and 3D to 1D profiles will be part of our future research.

Additional brains and their full cortex cortical layer labels will be acquired and used to train a new deep learning model. In future work, data from a larger set of brains (myelin- and Nissl-stained histology), along with their annotated cortical region annotations and layer labels, will be used to extensively test our pipeline and expand our results to create a layer segmentation of the entire cerebral cortex.

## Information Sharing Statement

Dataset and cortical layer segmentation results are available at: https://figshare.com/s/51a81a03003077c620e7?file=46337365. The CycleGAN and pix2pix source code can be downloaded from GitHub repository at https://github.com/junyanz/pytorch-CycleGAN-and-pix2pix. The nnU-Net source code can be accessed via this link: https://github.com/MICDKFZ/nnUNet/releases/tag/v2.2. The trained nnU-Net model can be found at https://figshare.com/s/0882f914e5bc57d2aa35?file=47998354.

## Supplementary information

The deep learning model and training details are provided in the supplementary information document.

## Supplementary Information

Below is the link to the electronic supplementary material.

Supplementary file 1 (pdf 277 KB)

## Data Availability

The segmentation results of the cerebral cortex laminar structure of the auditory cortex for one common marmoset monkey brain can be accessed via this link: https://figshare.com/s/51a81a03003077c620e7. The trained model can be accessed via this link: https://figshare.com/s/0882f914e5bc57d2aa35.

## Footnotes

1 https://pillow.readthedocs.io/en/stable/porting.html

2 https://github.com/ANTsX/ANTs/blob/master/Scripts/antsRegistrationSyN.sh

3 https://github.com/ANTsX/ANTs/wiki/Forward-and-inverse-warps-for-warping-images,-pointsets-and-Jacobians

4 https://github.com/MIC-DKFZ/nnUNet

## Declarations

All experimental procedures were approved by the Experimental Animal Committee of RIKEN, or by the Experimental Animal Committee of the National Center of Neurology and Psychiatry. The marmosets were handled in accordance with the Guiding Principles of the Care and Use of Animals in the Field of Physiological Science formulated by the Japanese Physiological Society.
