# Supplementary material for: A Deep Learning-based Pipeline for Segmenting the Cerebral Cortex Laminar Structure in Histology Images
Source: Neuroinformatics. 2024 Oct 17;22(4):745–61. doi: 10.1007/s12021-024-09688-0 (PMC11579130; doi:10.1007/s12021-024-09688-0)
Supplement: Supplementary file 1 — (pdf 277 KB) [file 12021_2024_9688_MOESM1_ESM.pdf]

Supplementary Information

1 The Core Image Segmentation Model

Image patches are made by the Patch Maker module and are then passed to an image segmentation model, called nnU-Net (Isensee et al., 2021). nnU-Net is an image segmentation framework developed for medical image processing; the core of the segmentation model is U-Net with some modifications. For example, the z-score normalization (Ulyanov et al., 2017), leaky ReLU activation function (Ulyanov et al., 2017) and deep supervision module (Li et al., 2022) were replaced or added to the original U-Net deep learning model. The final layer contains one convolution layer with a kernal size of 1×1 and seven feature maps, six feature maps per layer, and one feature map for the background. These feature maps then pass through a softmax activation function (Sharma et al., 2017) to produce the final multi-class segmentation mask image.

nnU-Net has a built-in module for cropping the large image to small patches, however, since, we developed our own Patch Maker module, the built-in module was disabled by simply changing the patch_size in the "nnUNetPlans.json" file to 512×512 before executing the "nnUNetv2_preprocess" command. Moreover, the batch_size value was also changed to 32 instead the lower number generated by nnU-Net.

2 Model Training Details

The model is trained using the sum of the cross-entropy loss (L_{CE}) (Zhang & Sabuncu, 2018) and Dice loss (L_{Dice}) (Drozdzal et al., 2016; Jadon, 2020). The lowest loss values for L_{CE} and L_{Dice} were -1 and 0, respectively. We expect the total loss L_{total} to decrease from positive infinity to as close as possible to -1 during the training phase. For the optimizer, the stochastic gradient descent with Nesterov momentum ($\mu = 0.99$) optimiser (Dozat, 2016) was used. The learning rate was changed to 0.005 (0.01 as the default value), and a new nnU-Net trainer was created based on the existing source code, and altered the maximum training epoch to 100.

Three brains were used as our image dataset for training, validation, and testing. The original nnU-Net train-val splitting module was overwritten by our own source code so that custom data splitting could be performed. Since the file name of each patch image has a brain ID associated with it, our Python source code reads the patch image file name and extracts the brain ID, then splits the training, validation, and

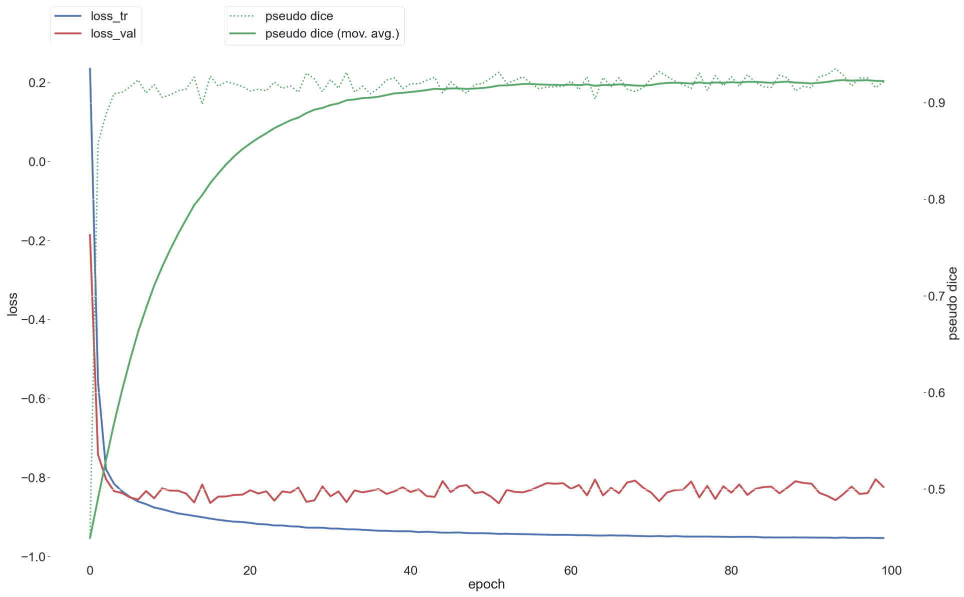

Fig. 1: Train-val Loss Plot

testing set based on that, so that 3-fold cross-validation can be performed. The minimum training loss and validation loss observed were -0.953 and -0.864, respectively. Fig. 1 shows the training and validation loss plot for one of the folds.

References

Dozat, T. (2016). Incorporating Nesterov Momentum into Adam. *The Proceedings of the 4th International Conference on Learning Representations*, 1–4.

Drozdzal, M., Vorontsov, E., Chartrand, G., Kadoury, S., Pal, C., Mateus, D., Peter, L., Bradley, A., Tavares, J. M. R. S., Belagiannis, V., Papa, J. P., Nascimento, J. C., Loog, M., Lu, Z., Cardoso, J. S., & Cornebise, J. (2016). The importance of skip connections in biomedical image segmentation. *Deep Learning and Data Labeling for Medical Applications*, 179–187.

Isensee, F., Jaeger, P. F., Kohl, S. A. A., Petersen, J., & Maier-Hein, K. H. (2021). nnU-Net: a self-configuring method for deep learning-based biomedical image segmentation. *Nature Methods*, *18*(2), 203–211.

Jadon, S. (2020). A survey of loss functions for semantic segmentation. *2020 IEEE Conference on Computational Intelligence in Bioinformatics and Computational Biology (CIBCB)*. https://doi.org/10.48550/arXiv.2006.14822

Li, R., Wang, X., Huang, G., Yang, W., Zhang, K., Gu, X., Tran, S. N., Garg, S., Alty, J., & Bai, Q. (2022). A Comprehensive Review on Deep Supervision: Theories and Applications. https://doi.org/10.48550/arXiv.2207.02376

Sharma, S., Sharma, S., & Athaiya, A. (2017). Activation functions in neural networks. *Towards Data Sci*, *6*(12), 310–316.

Ulyanov, D., Vedaldi, A., & Lempitsky, V. (2017). Instance Normalization: The Missing Ingredient for Fast Stylization.

Zhang, Z., & Sabuncu, M. R. (2018). Generalized Cross Entropy Loss for Training Deep Neural Networks with Noisy Labels. https://doi.org/10.48550/arXiv.1805.07836
